# The multifaceted role of astrocytes in regulating myelination

**DOI:** 10.1016/j.expneurol.2016.03.009

**Published:** 2016-09

**Authors:** Hülya Kıray, Susan L. Lindsay, Sara Hosseinzadeh, Susan C. Barnett

**Affiliations:** Institute of Infection, Inflammation and Immunity, Sir Graeme Davies Building, Glasgow Biomedical Research Centre, 120 University Place, University of Glasgow, Glasgow G12 8TA, United Kingdom.

**Keywords:** Astrocytes, Phenotypes, Oligodendrocytes, Microglia, Neurons, Myelination

## Abstract

Astrocytes are the major glial cell of the central nervous system (CNS), providing both metabolic and physical support to other neural cells. After injury, astrocytes become reactive and express a continuum of phenotypes which may be supportive or inhibitory to CNS repair. This review will focus on the ability of astrocytes to influence myelination in the context of specific secreted factors, cytokines and other neural cell targets within the CNS. In particular, we focus on how astrocytes provide energy and cholesterol to neurons, influence synaptogenesis, affect oligodendrocyte biology and instigate cross-talk between the many cellular components of the CNS.

## Highlights

• Astrocytes secrete factors that promote neuronal survival and synaptogenesis.
• Astrocyte-secreted factors influence oligodendrocyte precursor cell biology.
• Astrocytes can influence myelination, which involves the cross-talk by many cells within the CNS.
• Cytokines and chemokines produced by astrocytes can be potent regulators of myelination.

## 1 Introduction

Astrocytes were long considered secondary to neurons in central nervous system (CNS) function, and erroneously dismissed as “brain glue” (glia is the Greek term for glue). Research over the past two decades, however, has shown astrocytic roles extending to a range of brain functions far beyond basic physical and metabolic neuronal support (Sofroniew and Vinters, 2010). Astrocytic regulation of myelination was first hypothesised by Műller in 1904, who claimed that the demyelinating disease, Multiple Sclerosis (MS), was rooted in astrocytic dysfunction (Müller, 1904, Williams et al., 2007). Evidence has since continued to grow supporting the premise that astrocytes could be important in regulating myelination (Sofroniew and Vinters, 2010, Williams et al., 2007, Barnett and Linington, 2013, Moore et al., 2011).

**Fig. 1:**
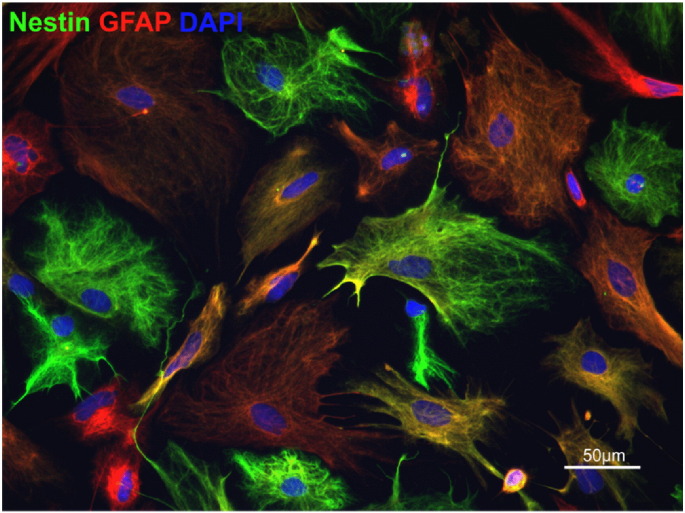
Expression of astrocyte reactivity markers. Rat neurosphere-derived astrocytes cultured on PLL-coated glass coverslips express the reactivity markers GFAP (green) and nestin (red).

Glial fibrillary acidic protein (GFAP) has been used extensively in the study of astrocytes. Increased GFAP expression has been associated with astrocyte reactivity in CNS lesions and is a pathological hallmark of disease and/or injury. Fig. 1 illustrates astrocytes immunolabelled with GFAP and nestin, another marker thought to label reactive astrocytes (Kamphuis et al., 2012). In experimental allergic encephalomyelitis (EAE), a widely used animal model of MS, where demyelination is induced by myelin antigens, administered together with adjuvant that contains bacterial components (Traugott and Lebon, 1988, Tsukada et al., 1991, Villarroya et al., 1996), GFAP expression was seen on more numerous and much larger astrocytic processes in chronic lesions compared to normal appearing white matter (Webster et al., 1985, Eng et al., 1971). Thus, the degree of GFAP immunoreactivity appears to reflect the level of reactive astrogliosis. This was reviewed in detail by Sofroniew and Vinters (2010), who described a continuum of phenotypic changes, that range from mild to severe, the latter resulting in glial scar formation (Sofroniew and Vinters, 2010, Nash et al., 2011a). Attempts have also been made to define the astrocyte phenotype in more detail along this continuum (Liberto et al., 2004). It has been suggested that mild astrogliosis is associated with astrocyte “activation” and severe astrogliosis is associated with “reactivity”, with the former promoting recovery of CNS function after injury and the latter walling off the injured area and preventing repair (Liberto et al., 2004). Although activated astrocytes have been associated with less detrimental effects on the CNS and reactive astrocytes as more damaging, it is clear that these properties are not all or nothing and reactive astrocytes can also be beneficial to CNS repair. Interestingly, in studies using GFAP null mice, it was seen that animals had abnormal myelination, non-myelinated axons in the optic nerve with an age related reduction in myelin thickness (Liedtke et al., 1996). Non-conservative mutations in the GFAP gene have also been linked to the white matter brain disorder, Alexander disease (Brenner et al., 2001, Li et al., 2002). Therefore, the evidence for direct or indirect astrocytic roles on re/myelination has been established both by *in vitro* and *in vivo* studies.

**Fig. 2:**
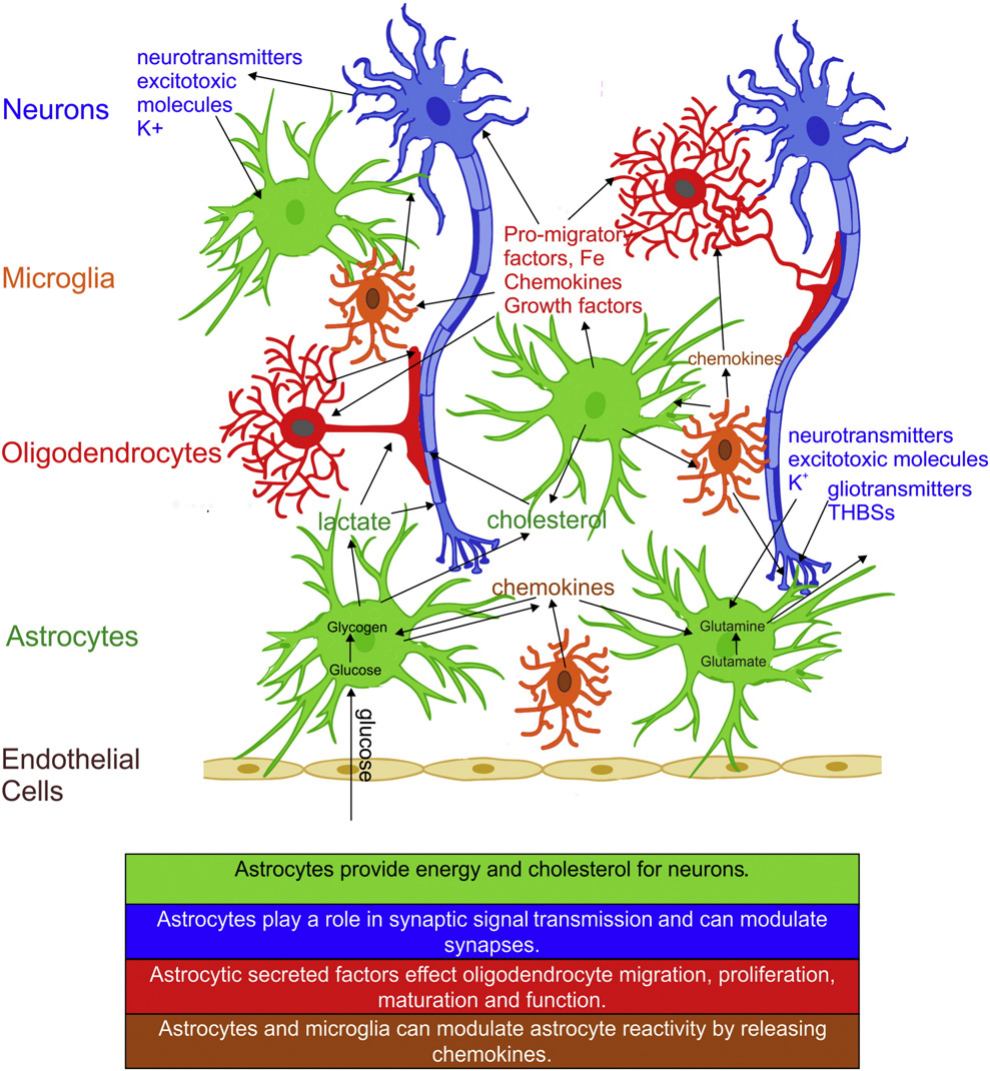
Astrocytic effects on re/myelination can be classified into 4 main groups. They contribute to re/myelination by: 1) Providing an energy source (lactate) and cholesterol for neurons. Glucose taken up by endothelial cells lining the blood brain barrier is later transferred to astrocytes which transform it to glycogen, which can then be used to produce lactate. 2) Playing a role in synaptic signal transmission by regulating the fluid, pH/ion (*e.g.* potassium, K ^+^), glio/neurotransmitter homeostasis and contributing to synapse modulation through secreted molecules, such as thrombospondins (THBSs). 3) Affecting the survival, proliferation and maturation of oligodendrocytes by secreting growth factors, some of which are regulated by iron homeostasis provided by astrocytes. Chemokines may also influence oligodendrocyte membrane ensheathment of axons. 4) Altering reactivity status through their release of chemokines/cytokines, which in turn affects the cross-talk between all neural cells including microglia.

Considering the limitations in the current treatments for demyelinating CNS diseases and injuries, it is crucial to identify other approaches to regulate myelination in search of novel strategies for repair. Astrocytes have been shown to promote myelination through their supportive roles on neuron survival and maintenance of neuronal activity, and their direct action on proliferation, differentiation and migration of oligodendrocytes (Fig. 2). This review will focus on the interaction of astrocytes with neural cells to synergistically promote myelination.

## 2 Astrocyte reactivity

### 2.1 Role of cytokines and chemokines

It is apparent that astrocytes can affect myelination under a range of normal and pathological conditions, but it is important to understand how this is regulated. Many molecules can trigger or even regulate astrogliosis, including large polypeptide growth factors and cytokines (John et al., 2003, Moore et al., 2011), mediators of innate immunity such as lipopolysaccharide (LPS) and other Toll-like receptor ligands (Farina et al., 2007), neurotransmitters (Bekar et al., 2008), purines, reactive oxygen species, and molecules related to hypoxia and glucose deprivation (Swanson et al., 2004). For the purpose of this review we will focus on cytokines and chemokines. Although these compounds are primarily considered in the context of chemotaxis in immune cells, here we will highlight their roles on astrocyte activation and reactivity. These molecules can be produced in an autocrine or paracrine fashion by various cell types in the CNS including neurons, oligodendrocyte lineage cells, microglia, pericytes and endothelial cells. Not only do these factors influence astrocyte phenotype but they also can affect a range of neural and immune cell types.

### 2.2 Astrocyte activation (mild astrogliosis): a pro-reparative phenotype?

**Fig. 3:**
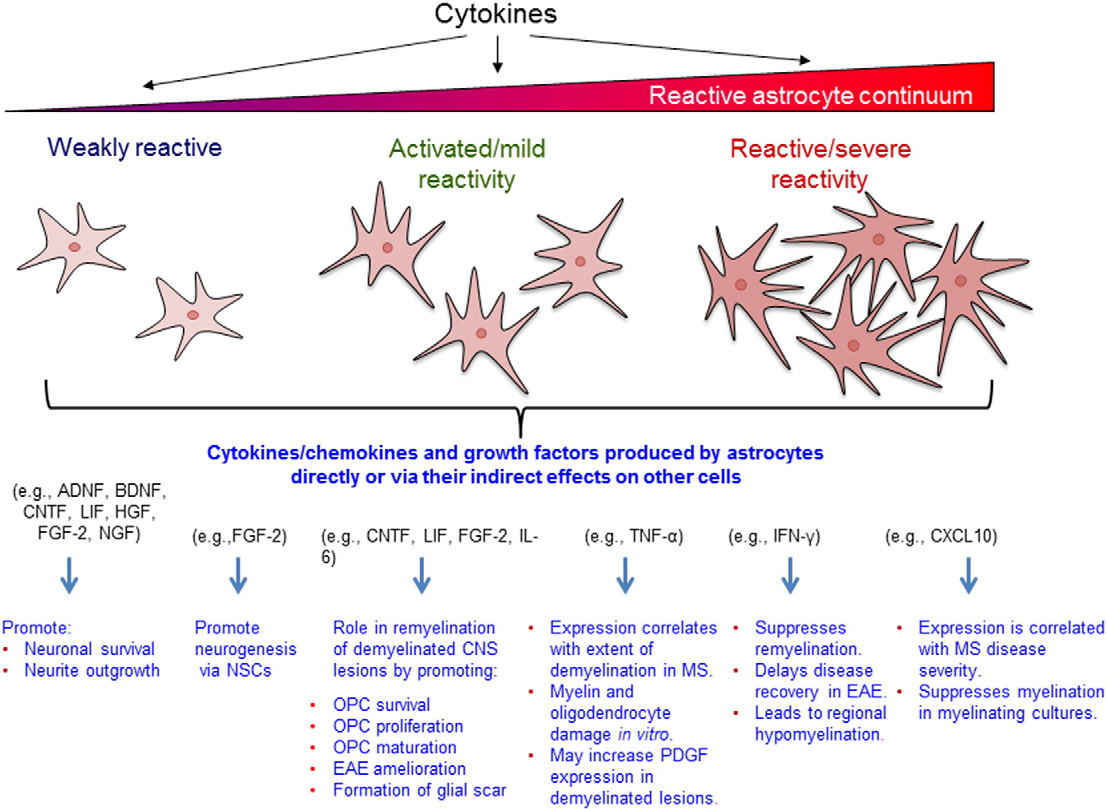
Simplified schematic of the effects of cytokine-activated astrocytes on re/myelination. Astrocytes can be influenced by various cytokines to change their reactivity status to one that falls within the continuum of phenotypes, namely more activated or reactive; both of which will secrete factors that can modulate myelination in a positive or negative way. Astrocytes with more severe reactivity present increased GFAP expression, proliferation rate, and cellular hypertrophy with a more stellate morphology. The milder “activated” astrocytes can secrete a range of factors including; neurotrophic factors, growth factors, and cytokines that will stimulate re/myelination by promoting neuronal survival, neurite outgrowth, neurogenesis, and/or oligodendrocyte precursor cell (OPC) survival, proliferation, and/or maturation. Conversely astrocytes that tend to have a more severe “reactive” phenotype, possibly induced by pro-inflammatory cytokines/CNS tissue damage, may secrete cytokines and chemokines that lead to myelin and oligodendrocyte damage *in vitro*, suppress remyelination, delay disease recovery in experimental autoimmune encephalomyelitis (EAE), and suppress myelination in myelinating embryonic rat mixed spinal cord cultures. However, these reactive scar forming astrocytes can also protect CNS tissue by preventing immune cells from invading and exerting a pro-inflammatory response and have been shown to even ameliorate EAE.

Astrocytes can be activated directly or indirectly. For example, in response to injury, microglia become activated and release the cytokine interleukin 1β (IL-1β, Herx et al., 2000), which is an early injury signal (Auron, 1998). The delay of astrocyte activation in mice lacking IL-1β, as well as in mice lacking IL-1 type 1 receptor suggests that microglial activation is necessary for astrocyte activation (Herx et al., 2000). It has also been suggested that ciliary neurotrophic factor (CNTF; a member of the IL-6 family of cytokines)treated astrocytes *in vitro* had a phenotype that was more supportive of CNS repair and thus are, by definition, activated (Albrecht et al., 2002, Albrecht et al., 2003, Albrecht et al., 2007). Under CNTF treatment, astrocytes upregulate expression of classical reactivity markers such as GFAP, vimentin, and clusterin, show cellular and nuclear hypertrophy, and increase their proliferation rate (Winter et al., 1995, Levison et al., 1996, Levison et al., 1998, Hudgins and Levison, 1998). There is a growing body of evidence for the promotion of neuronal survival by cytokine-activated astrocytes, potentially through secretion of various neurotrophic growth factors in the vicinity of neurons including nerve growth factor (NGF), brain-derived neurotrophic factor (BDNF), activity dependent neurotrophic factor (ADNF), hepatocyte growth factor (HGF), leukaemia inhibitory factor (LIF), fibroblast growth factor-2 (FGF-2) and CNTF (Schwartz and Nishiyama, 1994, Rudge et al., 1995, Uchida et al., 1998, Dreyfus et al., 1999, Messersmith et al., 2000, Albrecht et al., 2002; Fig. 3). Moreover, cultured spinal cord astrocytes, treated with CNTF, support the survival of a significantly greater number of ventral spinal motor neurons and promote neurite outgrowth better than unstimulated astrocytes (Albrecht et al., 2002). Other researchers have shown that cytokine-activated astrocytes can promote neurogenesis, possibly by stimulating the differentiation of neural stem cells (NSCs) residing in the subventricular zone and the dentate gyrus in adult animals (Liberto et al., 2004). Because these multipotent NSCs can migrate beyond their sites of origin and can later differentiate into oligodendrocytes, neurons and microglia, they have the potential to enhance recovery from CNS injury and disease.

Importantly, activated astrocytes have also shown positive effects on myelination. Our own investigations have shown that CNTF activated astrocytes can promote the percentage of myelinated fibres in CNS rat cultures (Nash et al., 2011b). Further evidence of this has been shown in mice infected with the A-59 strain of the mouse hepatitis virus (MHV-A59), an animal model for MS (Jordan et al., 1989**,** Messersmith et al., 2000). These animals have been shown to secrete increased levels of CNTF during the remyelination phase and CNTF mRNA is induced in the remyelinating regions in cells exhibiting astrocytic features (Albrecht et al., 2003). It is suggested that the increase in IL-1β levels at early stages of CNS pathology stimulates the induction of CNTF mRNA and protein in astrocytes (Stöckli et al., 1991, Guthrie et al., 1997, Dallner et al., 2002, Liberto et al., 2004), a phenomenon which appears to be important for remyelination (Herx et al., 2000). This could be due to FGF-2 signalling as CNTF treatment elevates astrocytic levels of *Fgf-2* mRNA significantly, whereas, IL-1 β shows no effect (Albrecht et al., 2003). Since FGF-2 can enhance OPC proliferation (Albrecht et al., 2003), it may produce more OPCs for subsequent myelination (Redwine et al., 1997, Messersmith et al., 2000). Moreover, if the gp130 receptor, the ubiquitous signal transducer for CNTF and all IL-6 family members, is genetically removed from astrocytes, astrocyte survival was poor, there was a reduction in the development of astrogliosis, and larger areas of demyelination formed with a greater pro-inflammatory T cell response (Haroon et al., 2011).

Therefore, CNTF seems to be an important cytokine involved in astrocyte reactivity and myelination. Interestingly, IL-1β can also stimulate the astrocytic production of another IL-6 family cytokine, LIF, (Aloisi et al., 1994), which has been shown to promote survival and differentiation of oligodendrocytes (Khan and De Vellis, 1994, Mayer et al., 1994, Bugga et al., 1998). LIF also decreases disease severity when exogenously administered in both chronic and relapsing-remitting EAE mice (Aloisi et al., 1994, Butzkueven et al., 2002, Ishibashi et al., 2006). Positive effects of LIF on the survival and maturation of oligodendrocytes also provides evidence for the positive roles of LIF on myelination (Khan and De Vellis, 1994, Mayer et al., 1994, Bugga et al., 1998). However, other pro-inflammatory cytokines such as tumour necrosis factor-alpha (TNF-α) and interferon-gamma (IFN-γ) have also been shown to potentiate reactive astrogliosis (Yong et al., 1991, John et al., 2003) as discussed below.

### 2.3 Astrocyte reactivity (severe astrogliosis): an inhibitory phenotype?

In contrast to their positive effects on myelination, astrocytes can also have a more detrimental effect on CNS repair *via* the secretion of chemokines/cytokines when in a more severe, reactive state (Fig. 3). One such cytokine is TNF-α, which has been shown to induce myelin and oligodendrocyte damage *in vitro* (Selmaj and Raine, 1988). *TNF-α* mRNA expression in MS plaques positively correlates with the extent of demyelination and has been shown to be present in microglia/macrophages and to a smaller percentage of astrocytes (Bitsch et al., 2000). On the other hand, studies have shown TNF-α expression is mainly associated with GFAP positive fibrous astrocytes in chronic active MS lesions at the lesion edge (Hofman et al., 1989) as well as foamy macrophages and endothelial cells (Selmaj et al., 1991). However, the fact that astrocytes appear as the major or minor TNF-α expressing cell types in MS lesions might be because astrocytes internalize the protein in a receptor-mediated manner (Aránguez et al., 1995, Kuhlmann et al., 2006) rather than producing it themselves as suggested by Hofman et al. (1989) and Bitsch et al. (2000). Moreover, it is possible that astrocytes require a longer period of time to become reactive upon injury and only produce TNF-α first at the lesion edge of acute MS plaques and later both at the lesion edge and in the lesion centre of chronic active plaques (Selmaj et al., 1991). *In situ* hybridisation for *TNF-α* mRNA has been detected in GFAP-positive astrocytes in mice suffering from pneumococcal meningitis (Izadpanah et al., 2014) which also suggests that astrocytes can indeed produce TNF-α in CNS pathologies.

Since TNF-α effects the maturation of oligodendrocytes (Cammer and Zhang, 1999), remyelination failure in the CNS lesions could be because TNF-α prevents the *in situ* differentiation of oligodendrocytes. Interestingly, direct cell contact between pre-oligodendrocytes (preOLs) and astrocytes has been shown to be a prerequisite for TNF to induce apoptosis in preOLs of rodent mixed glial cultures (Kim et al., 2011). Nevertheless, it is possible that TNF-α increases production of PDGF in demyelinated spinal cord lesions of MHV-A59-injected mice (Redwine and Armstrong, 1998, Frost et al., 2003) as suggested by the increase of PDGF-β transcription and PDGF-αβ protein levels in embryonic human astrocytes upon TNF-α treatment (Silberstein et al., 1996). Therefore, astrocytes might have a positive role on remyelination both by producing TNF-α and by secreting PDGF upon stimulation with TNF-α. PDGF could in turn support the survival and enhance the proliferation of OPCs in demyelinating lesions (Woodruff et al., 2004, Vana et al., 2007). Consequently, it is yet difficult to conclude whether reactive astrocytes associated with increased TNF-α levels in CNS lesions are predominantly stimulatory or inhibitory to remyelination.

IFN-γ, another cytokine found in MS plaques, has been reported to not only activate astrocytes, but is also expressed by reactive astrocytes and by immune cells that astrocytes have stimulated (Pulver et al., 1987, Traugott and Lebon, 1988, Miljkovic et al., 2007, Hashioka et al., 2009, Ionescu et al., 2011). Similar to TNF-α, IFN-γ has been shown to suppress remyelination and to delay disease recovery in transgenic EAE mice, where IFN-γ expression by astrocytes was stimulated temporally in the recovery stage (Lin et al., 2006). Astrocyte-directed expression of IFN-γ in transgenic mice has also resulted in regional hypomyelination and selective disruption of brain histogenesis, which led to ataxia and shorter life span (LaFerla et al., 2000). Furthermore, knocking down IFN-γ receptor expression in astrocytes three days before immunization suppressed EAE and demyelination by inhibiting inflammatory cell infiltration (Ding et al., 2015). These animals presented lower mean clinical scores even when the receptor silencing was initiated after disease onset or at disease peak (Ding et al., 2015). Despite the abovementioned evidence suggesting inhibitory roles for reactive astrocytes on myelination, Hindinger et al. (2012) have proposed that IFN-γ signalling in astrocytes is indispensable for the alleviation of EAE since levels of demyelination and axonal loss are increased during acute EAE in mice with an astrocytic expression of a dominant negative allele for IFN-γ receptor. Nevertheless, their approach blocked IFN-γ signalling in astrocytes without decreasing the expression of IFN-γ receptor, which would lower the levels of IFN-γ available for immunoregulatory cells; whereas, Ding et al. (2015) have knocked down the expression of the receptor itself. Therefore, blocking or lowering IFN-γ signalling in astrocytes with a carefully planned strategy might provide new disease-modifying treatments that will limit demyelination.

Reactive astrocytes also secrete C-X-C motif chemokine 10 (CXCL10, Ransohoff et al., 1993), particularly around active MS lesions (Omari et al., 2005, Carter et al., 2007). *Cxcl10* mRNA expression increases significantly during peak disease and decreases during the recovery phases in animal models of MS (Godiska et al., 1995, Glabinski et al., 1997, Fife et al., 2001). A direct effect of CXCL10 on the inhibition of myelination was shown in dissociated rat spinal cord cells plated on astrocytes treated with CXCL10 and its neutralizing antibody. In these experiments CXCL10 identified by microarray analysis, was upregulated in an astrocyte phenotype that was inhibitory to CNS myelination *in vitro*. Specifically, CXCL10 appeared to inhibit oligodendrocyte process extension (Nash et al., 2011b). Consequently, cytokines stand out as an important family of molecules to activate astrocytes and to initiate different forms of astrocyte reactivity that could be either beneficial or inhibitory for the CNS milieu in terms of re/myelination.

It should be noted that the secretion of such pro-inflammatory cytokines that can contribute to the lack of remyelination within MS plaques is not restricted to reactive astrocytes since other glial and inflammatory cell types will also secrete them. Moreover, the up regulation of such cytokines by reactive astrocytes can also be protective for CNS injury. For example, the astrocytic scar can restrict leukocyte migration from within areas of damaged tissue into the otherwise healthy non damaged CNS tissue in close proximity to the scar protecting it from immune mediated damage (Faulkner et al., 2004, Okada et al., 2006, Herrmann et al., 2008, Voskuhl et al., 2009).

## 3 Astrocytes provide energy and cholesterol

A vital supportive role played by astrocytes following injury is the provision of an energy source, which is important if axons are to be myelinated. This energy is metabolized from glucose which enters the brain *via* the endothelial cells lining the blood brain barrier (BBB), which are in close contact with astrocytes. Unlike endothelial cells, astrocytes biochemically transform glucose into glycogen, the principal source of stored energy in all cell types (Pellegri et al., 1996, Pfeiffer-Guglielmi et al., 2003). In addition, it has been suggested that astrocytes under low glucose concentrations can degrade stored glycogen into lactate which in turn increases extracellular lactate levels to provide energy for nearby axons when deprivation occurs after injury (Tekkök et al., 2005). The lactate derived from astroglial glycogen *via* glycolysis is transferred directly to the axon at the node of Ranvier (Hirrlinger and Nave, 2014). The importance of lactate during demyelination is only just emerging. Whether astrocytes maintain the energy levels of only axons as previously thought, or if this extends to oligodendrocytes as well, is an interesting concept. Oligodendrocytes are known to consume lactate at higher levels than other CNS cells, therefore making them an important user of any lactate production. Furthermore, promotion of myelination *via* oligodendrocytes has been shown when endogenous lactate is applied (Rinholm and Bergersen, 2012) therefore at least some astrocytic lactate production may be targeted to myelinating oligodendrocytes (Sánchez-Abarca et al., 2001, Rinholm et al., 2011). However, energy regulation in the CNS is more complex as recent evidence has shown that oligodendrocytes in turn can transfer glycolysis products such as lactate to axons *via* monocarboxylic acid transporters (MCT1, MCT2), which reside in internodal myelin and the axonal compartment (Fünfschilling et al., 2012).

Cholesterol is essential to every cell in the body as it is an important component of cellular membranes. In the CNS it is vital for normal brain development, is a precursor to many signalling molecules such as steroid hormones, and importantly, is a major structural component of myelin sheaths (Siegel et al., 1999). The BBB prevents the transport of either hepatic or dietary cholesterol, meaning that cholesterol must be derived by *de novo* synthesis within the CNS (Orth and Bellosta, 2012). Astrocytes are proposed to be one of the primary cellular sources of cholesterol (Pfrieger and Ungerer, 2011) and mediate its secretion by their expression of several apolipoproteins, molecules that bind cholesterol (Boyles et al., 1985, Lin et al., 1986, Xu et al., 2006, Kurumada et al., 2007). There is sufficient evidence to suggest that there is horizontal transfer of cholesterol (Boyles et al., 1985, Lin et al., 1986, Xu et al., 2006, Kurumada et al., 2007) with both astrocytes and oligodendrocytes producing cholesterol to maintain myelin sheath formation and neurons. This transfer between cells is critically relevant to neurodegenerative diseases, since the availability of cholesterol is thought to be an essential rate limiting factor to myelin production (May et al., 2004, Liu et al., 2010). Therefore, in addition to their roles in providing energy to neurons, astrocytes also emerge as important cholesterol-suppliers in the CNS, which is vital for myelination.

## 4 Astrocytes play a role in synaptic signal transmission and can modulate synapses

A further function of astrocytes is the removal of excitotoxic molecules from the extracellular space, thus supporting neuronal survival. They can actively remove excitotoxic glutamate and convert it to glutamine by increasing their levels of glutamate transporters and glutamine synthetase (Faden et al., 1989, Eng et al., 1997, Krum et al., 2002), thereby preventing neuronal cell death during brain pathology. Astrocytes can also release gliotransmitters such as glutamate, purine, GABA and D-serine into the synaptic cleft upon excitation by changes in neuronal synaptic activity and can thereby regulate neuronal excitability (Parpura et al., 1994, Bezzi et al., 1998, Mothet et al., 2000, Coco et al., 2003, Halassa et al., 2007). Neurotransmitter released from the neuronal synapse can reach adjacent astrocytes, stimulating increases in intracellular Ca^2 +^ concentrations, which then leads to the secretion of gliotransmitters (Porter and McCarthy, 1997). These regulatory molecules then can feedback to presynaptic nerve terminals to modulate synaptic neurotransmission (Araque et al., 1998). These observations have even given rise to the currently accepted ‘tripartite synapse’ hypothesis, where astrocytes form an active, integral regulatory component of the synapse (Araque et al., 1999, Halassa et al., 2007). Recently, electron microscopy has also shown microglia interacting with neuronal synapses (Tremblay et al., 2010) and playing a role in synapse maturation, synaptic remodelling, and synaptic activity (Ji et al., 2013). Evidence has shown that microglia secrete immune factors that play an important role in synaptic connections and illustrates the complexity of cross-talk between neural cells and the immune system. These researchers have suggested a change in name from tripartite synapse to the quad-partite synapse (Schafer et al., 2013, Wu et al., 2015).

In addition to playing a role in synaptic signal transmission, astrocytes can also modulate synapses. It has been demonstrated that astrocytes secrete molecules such as thrombospondins that might be required for the formation, function and pruning of developing synapses (Ullian et al., 2001, Christopherson et al., 2005). Both presynaptic and postsynaptic activity in purified rat retinal ganglion cultures have been enhanced in the presence of astrocytes; and immunohistochemistry of rat brain cryosections from various developmental stages have shown that glial generation precedes the appearance of synapses (Ullian et al., 2001). Astrocytes might also be functional in synaptic remodelling and pruning in healthy or diseased adult CNS (Barres, 2008). Because synaptic signal transmission can trigger astrocytes to secrete the cytokine LIF, which in turn increases the number of myelinated axons in dorsal root ganglion cultures co-cultured with oligodendrocytes (Ishibashi et al., 2006), the support and maintenance of healthy signal transmission appears important for the regulation of myelination.

Furthermore, astrocytes may contribute to synaptic transmission by supporting maintenance of the synaptic interstitial fluid by regulating ion, pH and transmitter homeostasis. Astrocyte processes contain transporters for potassium uptake and aquaporin 4 water channels (Nielsen et al., 1997, Rash et al., 1998, Amiry-Moghaddam et al., 2003, Solenov et al., 2004), which maintain the transmitter homeostasis of the synaptic interstitial fluid (Steinhäuser et al., 1994, Gundersen et al., 1996, Mennerick et al., 1996, Bergles and Jahr, 1997, Fujita et al., 1999). Connexin channels and connexin proteins are important candidates which play a role in the regulation of neuronal activity and survival. For example, astroglial connexins decrease neuronal excitability by removing extracellular potassium and glutamate; while also providing metabolic supply to neurons (Wallraff et al., 2006, Rouach et al., 2008, Froger et al., 2010, Pannasch et al., 2011). On the other hand, the elevation of connexin expression at lesion sites in CNS pathologies might also be associated with other, possibly protective, roles (Nagy et al., 1996, Koulakoff et al., 2008, Kuchibhotla et al., 2009, Mei et al., 2010, Karpuk et al., 2011). Studies using connexin knockout animals allude to the importance of connexins in promoting communication between astrocytes and between astrocytes and oligodendrocytes on myelin integrity. Myelin damage has been observed in Cx47, Cx43/Cx30 and Cx30/Cx47 single/double knockout mice (Menichella et al., 2003, Odermatt et al., 2003, Lutz et al., 2009, Tress et al., 2012). Interestingly, the level of oligodendrocyte gap junction connexins Cx47 and Cx32 were reduced both within and around lesions during early stages of inflammatory demyelination in EAE mice, (Traugott and Lebon, 1988, Tsukada et al., 1991, Villarroya et al., 1996, Meeuwsen et al., 2003). These mice also presented decreased expression of Cx43, the major astrocytic partner of Cx47, when spinal cord sections were analysed immunohistochemically (Markoullis et al., 2012). Cx43 expression was increased at late EAE stages, where remyelination was observed, leading to the suggestion that astrocytic protein Cx43 might play an important role in recovery from neuroinflammation (Markoullis et al., 2012).

## 5 Astrocytic effects on oligodendrocyte precursor cell (OPC) survival, proliferation and maturation

In the 1980s advancements were made on the ability to grow purified cultures of glial cells, with Raff and colleagues in particular developing techniques to purify OPCs from the optic nerve, a tissue devoid of neuronal cell bodies (Raff et al., 1983). With the development of these culture techniques it was shown that astrocytes play important roles in OPC differentiation (Noble and Murray, 1984, Noble et al., 1988, Raff et al., 1988, Noble et al., 1989) and in the rate of oligodendrocyte axonal ensheathment (Watkins et al., 2008). Further *in vitro* studies, where conditioned medium collected from primary astrocyte monolayers was incubated with other neural cells, showed enhanced neuronal survival, proliferation of OPCs, and protection of oligodendrocytes from stress (Noble and Murray, 1984, Yoshida et al., 1995, Yamamuro et al., 2003, Zhu et al., 2006, Arai and Lo, 2010). It is likely that astrocytes support OPC survival and proliferation by providing soluble factors such as platelet derived growth factor (PDGF) and FGF-2 (Bögler et al., 1990, Ferrara et al., 1988, Pringle et al., 1989).

Astrocytes are important providers of secreted growth factors, for both neuronal and glial proliferation and survival. For example, CNTF, although shown to be important in the activation of astrocytes, is constitutively expressed by white matter astrocytes, and is a key player in OPC survival and maturation *in vitro* and *in vivo* as discussed earlier (Stöckli et al., 1991, Dallner et al., 2002, Stankoff et al., 2002, Cao et al., 2010). CNTF has also been reported to enhance the migration of subventricular zone-derived progenitors (Vernerey et al., 2013), protect oligodendrocytes from apoptosis, and decrease myelin destruction in demyelinating pathological conditions (Linker et al., 2002). In studies when CNTF was injected subcutaneously at the remyelination phase of cuprizone-induced demyelination, an increase was seen in myelin oligodendrocyte glycoprotein (MOG) expression in the cerebral cortex (Salehi et al., 2013). Moreover, intraperitoneal injections of CNTF and intravenously transplanted mesenchymal stem cells that overexpress CNTF resulted in a reduced loss of neurons and disease severity, and increased neuronal functional recovery in EAE mice (Kuhlmann et al., 2006, Lu et al., 2009). However, in these experiments it is difficult to see if the effect is on the activation status of the astrocytes as discussed above, or the direct effect on the OPC.

Astrocytes have also been shown to exhibit a crucial role in OPC remyelination *via* their iron exporter ferroportin (Fpn) in mice, where focal demyelination was induced by the injection of lysophosphatidylcholine (LPC) into their spinal cords (Schulz et al., 2012). In these astrocyte-specific Fpn KO mice, fewer remyelinating axons and a reduction in OPC proliferation were observed following LPC-induced demyelination compared to control animals. This could either be due to direct effects on OPCs through limited iron supply or indirect effects *via* iron-deficient microglia, which expressed significantly lower levels of TNF-α and IL-1β when stimulated by LPS compared to control microglia (Schulz et al., 2012). Because the expression of FGF-2 and insulin-like growth factor-1 (IGF-1) was significantly upregulated by IL-1β and TNF-α, respectively, and the expression of transforming growth factor beta (TGF-β) was stimulated by IL-1β in purified astrocyte cultures, it has been suggested that iron-deprivation in the milieu would lower the expression of IL-1β and TNF-α in microglia and thus lead to reduced growth factor expression in astrocytes, which would in turn render OPC proliferation and possibly differentiation (Schulz et al., 2012).

## 6 Future directions in astrocyte research

As discussed throughout this review astrocytes can play important roles not just in myelination during development, but also in remyelination in adult tissue after CNS injury. Their reparative roles might be related to their level of reactivity, so it is important to identify markers that can define these different astrocyte phenotypes although at present these are not easy to define. One approach is to use microarrays. As described, Nash et al. (2011b) identified CXCL10 as inhibitory to myelination, but others using a lower-scale cDNA array that contained probes for cytokines, chemokines, growth factors and their receptors have identified other pro-inflammatory cytokines including TNF-α, IL-1β, or IFN-γ (Meeuwsen et al., 2003). Another large array was carried out on GFP-astrocytes purified at various time points after the onset of two models of disease, namely ischemic stroke (middle cerebral artery occlusion) and neuroinflammation induced by LPS injection (Zamanian et al., 2012). The resulting data suggested that astrocytes could present different mRNA expression profiles depending on the insult despite the presence of reactive gliosis in both types of CNS damage. However, although there was upregulation of a core set of genes (lipocalin 2 and serpina3n), it was clear that changes in the astrocyte after injury are highly heterogeneous, and that changes in astrocyte activity may depend on the injury type. Hopefully, more specific markers of “good” or “bad” astrocytes for CNS repair will be identified and allow more specific identification of how these astrocytes influence repair.

## 7 Summary and conclusions

There is abundant evidence to suggest that astrocytes contribute to re/myelination mainly by:

1) Providing the right conditions for neurons to myelinate by i) supplying neurons with energy and cholesterol, ii) removing excitotoxic molecules from the extracellular environment, and iii) regulating the fluid, ion, pH, and neuro/gliotransmitter homeostasis.

2) Playing a role in the survival, proliferation, maturation and function of oligodendrocytes and the migration of OPCs into the lesioned areas in the CNS.

3) Influencing microglia.

The manner by which astrocytes affect myelination can often be seen to correlate with its level of reactivity. Astrocyte reactivity can be induced by the milieu of cytokines present after injury, which can be beneficial or inhibitory in re/myelination depending on the context and severity of the injury. Due to the lack of gliosis-specific markers, there are currently no clear guidelines which allow different astrocytic reactivity phenotypes to be classified. However, if markers can be identified that classify the continuum of astrocyte phenotypes, it may aid in the design of new treatments targeting phenotypes that are more suited to regeneration and remyelination, and therefore benefit in the treatment of demyelinating diseases.
